# Sexual and bladder dysfunction in male ketamine abusers: A large-scale questionnaire study

**DOI:** 10.1371/journal.pone.0207927

**Published:** 2018-11-28

**Authors:** Stephen She-Dei Yang, Mei-Yu Jang, Kau-Han Lee, Wen-Tsang Hsu, Yi-Chu Chen, Wan-San Chen, Shang-Jen Chang

**Affiliations:** 1 Department of Urology, Taipei Tzu Chi Hospital, New Taipei, Taiwan; 2 School of Medicine, Buddhist Tzu Chi University, Hualien, Taiwan; 3 Department of Urology, Kaohsiung Municipal Hsia-Kang Hospital, Kaohsiung City, Taiwan; 4 Division of Urology, Department of Surgery, Chi Mei Medical Center, Tainan City, Taiwan; 5 Department of Urology, Keelung Hospital, Ministry of Health and Wealthfare, Keelung City, Taiwan; Medizinische Universitat Wien, AUSTRIA

## Abstract

**Purpose:**

To evaluate the prevalence of lower urinary tract symptoms (LUTS) and erectile dysfunction (ED) in the illicit male ketamine abusers (KA).

**Materials and methods:**

The male street KAs caught by policemen and patients visiting urologic clinics were invited to answer a structured questionnaire including demographic data, illicit drug use related details (duration, frequency, dosage and abstinence status), international prostate symptoms score (IPSS), interstitial cystitis symptoms and problem index (ICSI and ICPI) and International index of erectile function (IIEF-5). Erectile dysfunction was defined as IIEF-5 ≦21.

**Results:**

Finally, we included 1056 participants (993 street, 63 hospital KAs) with a mean age of 27.4 ±6.2 years. ED presented in 30.8% of all KAs. and Hospital KAs were more subject to having ED than street KAs (69.6% vs. 28.0%, p<0.01). Multi-variate analysis revealed that risk factor for male ED were age ≧30 years (OR = 1.765). Subgroup analysis on male street KAs disclosed that abstinence ≧3 months is a protective factor for ED. Lower urinary tract symptoms (ICSI+ICPI ≧12) was prevalent in KAs and multivariate analysis disclosed that significant risk factors for LUTS (ICSI+ICPI ≧12) were age ≧30 years, duration ≧24 months and co-use of other illicit drugs.

**Conclusions:**

Male ED and LUTS were frequently observed in the ketamine abusers. We suggested that street ketamine abuse should be considered in young men presented with ED and LUTS in the clinics.

## Introduction

Ketamine is an antagonist of the N-methyl-D-aspartic acid (NMDA) receptor complex and is mainly used for anesthesia. Also, ketamine is also being studied as a treatment for mental disorders (ie. depression[1] and pain[2]). However, it is now frequently used as a recreation drug due to its dissociative effect. In Taiwan, ketamine is classified as grade III illicit drug and was second to ecstasy as the most commonly consumed illegal drugs among young people[3]. Illicit ketamine use is associated with urological burden such as ketamine cystitis and erectile dysfunction[4–6]. Large surveys revealed 20–30% of ketamine abusers suffered with lower urinary tract symptoms due to ketamine cystitis (KC).

Erectile dysfunction (ED) is characterized by an inability to achieve and maintain penile erection for sexual performance. The relationship between illicit use of ketamine and male sexual function was less clear. Long-term administration of ketamine induced erectile dysfunction in rats. [7]. Suppiah et al[5] reported that half of 127 Malaysians, mostly ethnic Chinese, using ketamine and polydrugs admitted erectile dysfunction. Recently, animal study showed that ketamine could induce erectile dysfunction in male rats[8]. Up to date, there was no large scale study using validated questionnaire to survey erectile dysfunction in male KAs.

A group of experts in Taiwan met and reached a general consensus on the recommended assessment of ketamine associated uropathy[9]. International Prostate Symptoms Score (IPSS), Interstitial Cystitis Symptoms and Problem Index (ICSI and ICPI) were recommended to assess LUTS in KAs because these questionnaires have been widely used by Taiwanese urologists[10]. Herein, we reported the prevalence of erectile dysfunction and LUTS among street and hospital KAs. Risk factors of erectile dysfunction and LUTS were analyzed and reported.

## Materials and methods

The study was approved by ethic committee of Taipei Tzu Chi Hospital, Buddhist Tzu Chi Medical Foundation. Ketamine is graded as level III illicit drug in Taiwan. Street KAs caught by policemen were requested to attend an educational course for 6–8 hours in Taiwan[3]. If there is concomitant use of higher level of illicit drugs (i.e. Heroin, morphine, opiate, cocaine, amphetamine, NMDA, and marijuana), they will be detained in a Drug Abstention and Treatment Center for detoxication for at most 2 months[11]. Therefore, the study included only those with ketamine and those with higher level of illicit drug were not included in the study. The study participants comprised men aged≥ 18 years. During the educational courses, all participants were given a verbal explanation of the study. After the participants completed the written informed consents, they were invited to answer a structured questionnaire anonymously, which included demographic data, history of alcohol consumption, smoking, other illicit drug abuse in addition to ketamine (i.e.. Heroin, morphine, opiate, cocaine, amphetamine, NMDA, and marijuana etc.), visual pain score (0–10), International Prostate Symptom Score (IPSS), brief form International Index of Erectile Function (IIEF-5) and the Interstitial Cystitis Symptom Index (ICSI) and Problem Index (ICPI)[10] questionnaires. The same questionnaires were answered by patients visiting urologic clinics for management of LUTS.

### Dosage of ketamine, duration and abstinence status of ketamine

Participants were then asked to answer the average daily dosage of ketamine they used (g/day), the route of ketamine use (oral ingestion, inhalation, snorting or smoking cigarettes with ketamine), and the duration of ketamine use (months). The participant was asked whether they had quit the ketamine or not. If the participant had quit ketamine, the period of ketamine abstinence was recorded (months).

### Erectile dysfunction

The sexual function was evaluated with the IIEF-5. Response options are based on rating scales from 1 to 5. The responses are summed resulting in a total IIEF-5 score ranging from 5 to 25. A score of 21 or lower is regarded as having erectile dysfunction (ED). ED can be classified into five severity grades: absence of ED (IIEF-5 score 22–25), mild (17–21), mild to moderate (12–16), moderate (8–11), and severe (5–7)[12]

### Lower urinary tract symptoms

The lower urinary tract symptoms were evaluated with the International Prostate Symptom Score (IPSS), Interstitial Cystitis Symptom Index (ICSI) and Problem Index (ICPI)[8] An IPSS of 8 or more is regarded as abnormal. The original goal of ICSI and ICPI I questionnaire is to evaluate and diagnose patients with interstitial cystitis and each questionnaire contains four questions related to urinary and pain symptoms. A score of (ICSI + ICPI) ≥12 points is regarded as abnormal. The Symptom Index included four symptoms: 1. whether the patient feels the need to urinate with little or no warning 2. has to urinate more frequently than every 2 hours, 3. needs to get up during the night to urinate, and 4. has pain in the bladder. The Problem Index evaluate: 1. urinary frequency during the day, 2. urinary frequency at night, 3.the need to urinate with little or no warning, and 4. burning, pain, discomfort, or pressure on the bladder.

### Statistics

Data were expressed as mean ± standard deviation and were analyzed by commercial statistical software (SAS, version 9.4, USA). Demographic and voiding parameters were compared via an independent sample t test (continuous demographic variables), a χ2 test (nominal data), and a Mann-Whitney U test (ordinal data). Multivariate logistic regression was used to evaluate the risk factors, ie. Age, cigarette smoking (yes vs. no), alcohol consumption (yes vs. no), dosage of ketamine (dosage >2 or < = 2 g), and duration of illicit ketamine use (months), route of ketamine administration (smoking ketamine cigarette vs. others), Co-use of other illicit drugs (yes vs. no) and abstinence period of illicit ketamine for male sexual dysfunction (IIEF-5 < = 21). For all tests, a p-value of less than 0.05 was regarded as significant.

## Results

Between 2016 and 2017, 993 street KAs and 63 hospital KAs were enrolled. Mean age of the subjects are 22.76 ± 6.4 years. Table 1 summarizes the demographic data and results of questionnaire. The mean duration and dosage of illicit ketamine use is 31.6±37.3 months and 2.20±2.89 g/day. KAs enrolled from hospitals had higher average daily dose and longer period of using of drugs, higher score of LUTS (IPSS, ICSI, ICPI), higher pain score, and lower score of IIEF-5 than those from street.

**Table 1:** Demographic data and results of questionnaires in street and hospital male ketamine abusers.

	Total	Street Abusers	Hospital Abusers	*p*-value
N	1056	993 (94.03%)	63 (5.97%)	
Age in years	27.76±6.40	27.59±6.30	29.82±7.24	0.0078*
Duration (months)	31.64±37.33	30.36±35.85	51.41±51.79	0.0078*
Daily dose (g)	2.20±2.89	2.04±2.73	4.11±3.98	<0.0001*
IPSS score	3.67±6.37	2.71±4.43	17.40±11.89	<0.0001*
ICSI score	2.023±3.40	1.50±2.26	10.33±6.53	<0.0001*
ICPI score	1.90±3.76	1,37±2.86	10.11±6.00	<0.0001*
IIEF-5 score	22.10±4.13	22.35±3.97	18.76±4.62	<0.0001*
Pain score	0.58±1.67	0.36±1.15	4.10±3.70	<0.0001*

*: a p-value of less than 0.05 was regarded as significant.

### Erectile dysfunction

Complete IIEF-5 was replied in 804 male KAs of whom 30.8% with IIEF-5 ≦21 were regarded as having erectile dysfunction (Table 2). ED was more commonly frequently observed in hospital than street KAs (69.6% vs. 27.9%, p<0.001). Univariate analysis revealed that age ≧30 years and duration of ketamine use were significant risk factors for ED in ketamine abuser. Also, abstinence of ketamine is a protective factor for erectile function among the participants. However, average daily dose, route of ketamine use, cigarette and alcohol consumption did not pose significant effects on ED. Multi-variate analysis disclose that the only risk factor for ED in all male KAs were ≧30 years (Table 3). Subgroup analysis on male street KAs disclosed that age≧30 years is a risk factor of ED and abstinence ≧3 months is a protective factor(Table 4).

**Table 2:** Grade of erectile dysfunction by IIEF-5 in all ketamine abusers.

	All	Street KA	Hospital KA	*p*-value
N	804	748	56	
Normal (22–25)	556 (69.15%)	539 (72.06%)	17 (30.36%)	<0.0001*
Mild (17–21)	176 (21.89%)	155 (20.72%)	21 (37.50%)	
Mild to moderate (12–16)	39 (4.85%)	26 (3.48%)	13 (23.21%)	
Moderate (8–11)	15 (1.87%)	11 (1.47%)	4 (7.14%)	
Severe (≦7)	18 (2.24%)	17 (2.27%)	1 (1.79%)	

*: a p-value of less than 0.05 was regarded as significant.

**Table 3:** Risk factors of IIEF-5 < = 21 in all male ketamine abusers.

Variable	IIEF-5>21	IIEF-5< = 21	cOR	aOR^$^
Age in years				
<25	214 (76.16%)	67 (23.84%)	1	1
25–30	176 (71.84%)	69 (28.16%)	1.252 (0.847–1.851)	1.153 (0.728–1.827)
> = 30	165 (59.57%)	112 (40.43%)	2.168 (1.506–3.121)*	2.092 (1.367–3.200)*
Duration (months)				
< = 12	225 (73.29%)	82 (26.71%)	1	1
12–24	52 (61.90%)	32 (38.10%)	1.689 (1.016–2.806)*	1.535 (0.897–2.625)
> = 24	143 (64.71%)	78 (35.29%)	1.497 (1.029–2.176)*	1.296 (0.865–1.942)
Missing	135	56		
Average daily dose				
<2 (g)	340 (68.14%)	159 (31.86%)	1	
> = 2 (g)	130 (68.78%)	59 (31.22%)	0.971 (0.677–1.392)	
Missing				
Period of abstinence in months				
0 Still use	52 (61.90%)	32 (38.10%)	1	1
<3	55 (67.07%)	27 (32.93%)	0.798 (0.422–1.509)	0.836 (0.417–1.673)
3–6	68 (77.27%)	20 (22.73%)	0.478 (0.246–0.930)*	0.507 (0.239–1.072)
> = 6	336 (69.14%)	150 (30.86%)	0.725 (0.449–1.173)	0.822 (0.484–1.398)
Missing				
Route of ketamine use				
Smoking ketamine cigarette	490 (69.31%)	217 (30.69%)	1	
Others (snorting or oral ingestion)	29 (65.91%)	15 (34.09%)	1.168 (0.614–2.223)	
Missing	36	16		
Co-use of other illicit drugs				
No	487 (70.17%)	207 (29.83%)	1	
Yes	40 (59.70%)	27 (40.30%)	1.588 (0.949–2.657)	
Missing	28	14		
tobacco smoking				
No	35 (66.04%)	18 (33.96%)	1	
Yes	519 (69.76%)	225 (30.24%)	0.843 (0.467–1.520)	
Missing	1	5		
Alcohol				
No	210 (67.74%)	100 (32.26%)	1	
Yes	340 (70.25%)	144 (29.75%)	0.889 (0.654–1.210)	
Missing	5	4		

*p<0.05.

**Table 4:** Risk factors of IIEF < = 21 among only street ketamine abusers.

Variable	IIEF>21	IIEF< = 21	cOR	aOR^$^
Age in years				
<25	210 (78.07%)	59 (21.93%)	1	1
25–30	169 (72.84%)	63 (27.16%)	1.327 (0.881–1.997)	1.310 (0.855–2.007)
> = 30	159 (64.63%)	87 (35.37%)	1.947 (1.319–2.875)*	2.015 (1.341–3.026)*
Duration (months)				
< = 12	222 (74.75%)	75 (25.25%)	1	
12–24	52 (65.00%)	28 (35.00%)	1.594 (0.939–2.705)	
> = 24	135 (69.95%)	58 (30.05%)	1.272 (0.849–1.905)	
Missing	129	48		
Average daily dose				
<2 (g)	337 (70.21%)	143 (29.79%)	1	
> = 2 (g)	120 (73.17%)	44 (26.83%)	0.864 (0.581–1.285)	
Missing	81	22		
Period of abstinence in months				
0 Still use	50 (64.94%)	27 (35.06%)	1	1
<3	53 (69.74%)	23 (30.26%)	0.804 (0.408–1.582)	0.735 (0.370–1.459)
3–6	68 (80.95%)	16 (19.05%)	0.436 (0.212–0.893)*	0.394 (0.190–0.814)*
> = 6	328 (72.09%)	127 (27.91%)	0.717 (0.430–1.195)	0.668 (0.398–1.121)
Missing	39	16		
Route of ketamine use				
Smoking ketamine cigarette	484 (71.92%)	189 (28.08%)	1	
Others(snorting or oral ingestion)	23 (74.19%)	8 (25.81%)	0.891 (0.392–2.026)	
Missing	31	12		
Co-use of other drugs				
No	477 (72.05%)	185 (27.95%)	1	
Yes	36 (75.00%)	12 (25.00%)	0.859 (0.438–1.688)	
Missing	25	12		
tobacco smoking				
No	33 (66.00%)	17 (34.00%)	1	
Yes	505 (72.45%)	192 (27.55%)	0.738 (0.402–1.356)	
Alcohol				
No	202 (70.38%)	85 (29.62%)	1	
Yes	332 (72.97%)	123 (27.03%)	0.880 (0.635–1.221)	
Missing	4	1		

*p<0.05.

### Lower urinary tract symptoms

LUTS (ICSI+ICPI ≧12) is prevalent in KAs although these participants are young men and are more frequently observed in the hospital KAs than street KA (77.6% vs. 6.7%, p<0.001). Table 5 lists the univariate and multivariate analysis for risk factor of lower urinary tract symptoms. Univariate analysis revealed that age ≧30 years, duration of ketamine use ≧24 months, average dosage ≧2 g and co-use of other illicit drugs were significant risk factors for ED in ketamine abuser. Multivariate analysis disclosed that significant risk factors for LUTS (ICSI+ICPI ≧12) were age ≧30 years, duration ≧24 months and co-use of other illicit drugs. Average dosage ≧2 g also pose impact on LUTS despite insignificant on multivariate anaylsis.

**Table 5:** Risk factors (ICSI+ICPI> = 12) in all male ketamine abusers.

Variable	ICSI+ICPI<12	ICSI+ICPI> = 12	cOR	aOR^$^
Age in years				
<25	275 (93.54%)	19 (6.46%)	1	1
25–30	221 (91.32%)	21 (8.68%)	1.375 (0.721–2.622)	1.422 (0.621–3.256)
> = 30	232 (82.27%)	50 (17.73%)	3.119 (1.788–5.441)*	3.805 (1.862–7.777)*
Missing	152	12		
Duration (months)				
< = 12	348 (95.08%)	18 (4.92%)	1	1
12–24	92 (92.93%)	7 (7.07%)	1.471 (0.596–3.628)	0.982 (0.364–2.649)
> = 24	221 (81.55%)	50 (18.45%)	4.374 (2.487–7.691)*	2.061 (0.999–4.253)
Missing	219	27		
Average daily dose				
<2 (g)	568 (94.35%)	34 (5.65%)	1	1
> = 2 (g)	188 (77.69%)	54 (22.31%)	4.799 (3.031–7.600)*	3.721 (1.971–7.025)*
Missing	124	14		
Route of ketamine use				
Smoking ketamine cigarette	657 (90.50%)	69 (9.50%)	1	
Others (snorting or oral ingestion)	180 (88.24%)	24 (11.76%)	1.270 (0.776–2.078)	
Missing	43	9		
Co-use of other drugs				
No	688 (91.13%)	67 (8.87%)	1	1
Yes	144 (84.71%)	26 (15.29%)	1.854 (1.139–3.018)*	3.285 (1.587–6.801)*
Missing	48	9		
Smoking				
No	55 (93.22%)	4 (6.78%)	1	
Yes	823 (89.65%)	95 (10.35%)	1.587 (0.563–4.477)	
Missing	2	3		
Alcohol				
No	322 (89.20%)	39 (10.80%)	1	
Yes	551 (90.48%)	58 (9.52%)	0.869 (0.566–1.334)	
Missing	7	5		

* p<0.05.

### Correlations between sexual and bladder dysfunction in all ketamine abusers

**Fig 1:**
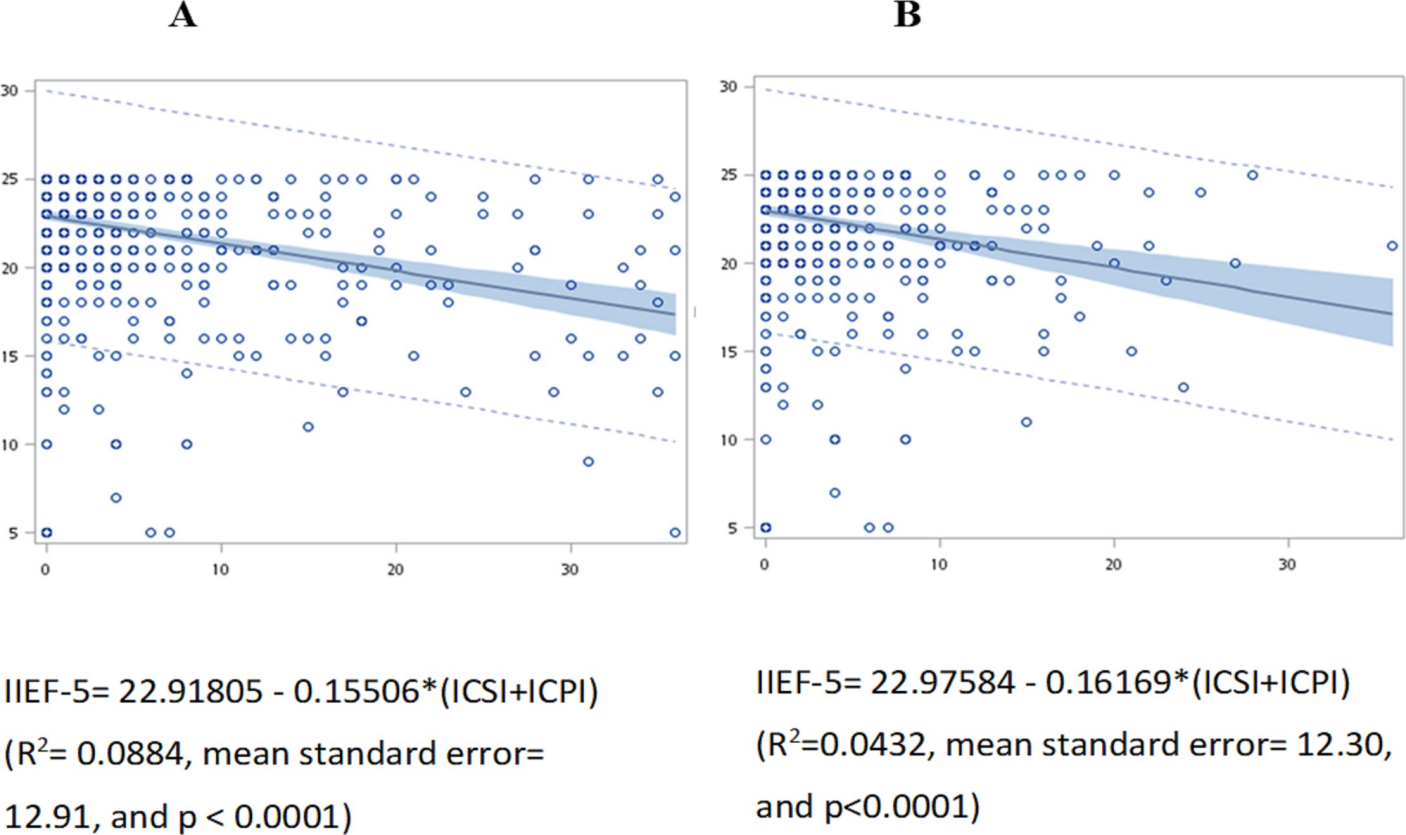
**Correlations between sexual (IIEF-5, Y axis) and bladder dysfunction (ICSI+ICPI, X axis) in 734 male street and hospital ketamine abusers (1A) and 681 street ketamine abusers (1B)**.

Sum score of ICSI plus ICPI was negatively associated with IIEF-5 in all male KAs (p<0.0001, Fig 1A) and male street KAs (p<0.0001, Fig 1B).

## Discussion

Up to date, this is the largest case series to report sexual dysfunction and lower urinary tract symptoms in male illicit ketamine abusers which comprised those caught by policemen or visiting urologic clinics for managements of LUTS. Compared to street KAs, hospital KAs had higher average daily dose, longer period of ketamine abuse, higher score of LUTS and lower score in sexual function (Table 1). Male erectile dysfunction as defined by IIEF-5≦21 was reported in 30.8% of all KAs (Table 2), which was higher than the reported prevalence of healthy Taiwanese young men in which were 11.9% and 17.6%, respectively[11, 13]. Male ED was more frequently observed in hospital than street KAs (69.6% vs. 27.1%, p<0.0001) especially more mild to moderate ED was observed. Though most male street KAs were graded as mild ED, about 1/3 of male hospital KAs had higher grade of ED (Table 2). Recently, Suppiah et al used an yes or no question to define ED and reported that ED was present among half of Malaysians who used ketamine and poly-drugs[14]. In the ketamine-treated rats, long-term ketamine administration caused significantly decreased erectile responses, decreased smooth muscle content, reduced nNOS expression, increased iNOS content and increased corpus cavernosum apoptosis when compared with controls[7]. This may partly explain the reasons why ketamine could cause erectile dysfunction in young men. Similarly, illicit use of amphetamine was associated with erectile dysfunction[11]. High prevalence of erectile dysfunction in ketamine, amphetamine and other substance abuse may partly explain the significant misuse of sildenafil in London nightclubs[15].

Higher average daily dose and longer duration of ketamine abuse may partly explain the reasons of male ED in hospital KAs (Table 1) Univariate analysis disclosed risk factors of male ED were age ≧30 years and duration of abuse ≧12 months, while ketamine abstinence was a protective factor (Table 3). Multiple variate analysis showed that only age ≧30 years was risk factors for male ED. The reason why age as a risk factor of ED in KAs was unknown yet, though older age may be associated with longer duration and higher dose of ketamine abuse. Further studies on the age and effects of ketamine should be explored. Since our subgroup analysis on male street KAs disclosed that risk factors for ED were age≧30 years and abstinence ≧3 months is a protective factor(Table 4), more studies are required to prove that abstinence from ketamine does improve erectile function. Among these young participants, alcohol, cigarette smoking, route of ketamine administration and co-use of other illicit drugs did pose significant effect on ED.

Significant risk factors for having LUTS were older age, longer duration of ketamine use, and higher dosage of ketamine use which were in line with previous studies. Co-use of other illicit drugs also pose significant effects on LUTS.(Table 5) Previous case reports mostly enrolled participants of high dose (≧2 g) and long duration of ketamine abuse[16–18]. The relatively lower prevalence of LUTS among street KAs in the current study may be due to difference in questionnaire and duration of drug abuse. Our results on LUTS were similar to the reports by Chen et al [19]who used the same questionnaire to detect LUTS in KAs. They reported that among 143 participating ketamine users, 25 (17.5%) had LUTS. However, Chen et al did not report the LUTS in 118 street and 25 hospital ketamine abusers separately. Therefore, the true prevalence of LUTS in street KAs may be much lower. Shorter duration of street KAs in the current study (mean 32 months) may partly explain the lower incidence of LUTS in this study than Chen’s series (6.7% vs. 52%) in which a mean duration of 4 years was observed [1]. The significant association between erectile dysfunction and LUTS in male ketamine abusers can be attributed to longer duration and higher dose of ketamine which may have direct effects to both bladder and erectile functions.

The side effect of street ketamine may differ from the prescribed medical ketamine. The most commonly reported side effects associated with medical use of ketamine are psychiatric disorder, hypertension, nausea and vomiting[20]. Further studies are warranted to explore the differences in components between the street ketamine and medical-use ketamine.

Main limitation of the study lies in the volunteer filling in the questionnaire by the street KAs attending an educational course organized by the government. The variable dosage and duration of ketamine used within the period may lead to a significant bias. The self-reported dosage and a lack of objective parameters might result in measurement bias. Also, the study is lacking of normal control and whether ED or LUTS are more prevalent in ketamine abuser remained lacking of strong evidence. We are now conducting a large-scale study to evaluate the lower urinary tract function including erectile function and lower urinary tract symptoms among the healthy young men. Hoperfully, we can report the data in the near future. However, through the large scale study and comparisons between hospital KAs, the trend of more sexual dysfunction and LUTS in higher dose and longer duration abusers were confirmed indirectly. Secondly, we used the questionnaire to diagnose LUTS instead of using the more objective measurements, such as bladder diary and uroflowmetry. However, the large scale study made these diagnostic tests not feasible and a questionnaire (ICSI/ICPI) was used under the experts’ consensus. Thirdly, the comorbid psychiatric diseases were not recorded. Finally, the participants were not included through random sampling and therefore we enrolled a large number of study participants to overcome the bias.

### Conclusions

Male sexual dysfunction and lower urinary tract symptoms was frequently observed in the street and hospital ketamine abusers. We suggested that street ketamine abuse should be considered in young men presented with ED and LUTS in the clinics.

### IRB

The study was approved by ethic committee of Taipei Tzu Chi Hospital, Buddhist Tzu Chi Medical Foundation. **IRB: 04-XD32-091**

## Supporting information

S1 File (XLS)
